# Extra Virgin Olive Oil Prevents the Age-Related Shifts of the Distribution of HDL Subclasses and Improves Their Functionality

**DOI:** 10.3390/nu13072235

**Published:** 2021-06-29

**Authors:** Alyann Otrante, Amal Trigui, Roua Walha, Hicham Berrougui, Tamas Fulop, Abdelouahed Khalil

**Affiliations:** 1 Department of Medicine, Faculty of Medicine and Health Sciences, University of Sherbrooke, Sherbrooke, QC J1H 4N4, Canada; alyann.otrante@usherbrooke.ca (A.O.); Amal.trigui@usherbrooke.ca (A.T.); Roua.walhap@usherbrooke.ca (R.W.); Hicham.berrougui@USherbrooke.ca (H.B.); Tamas.fulop@usherbrooke.ca (T.F.); 2 Department of Biology, Polydisciplinary Faculty, University Sultan Moulay Slimane, 23000 Beni Mellal, Morocco

**Keywords:** atherosclerosis, HDL, EVOO, cholesterol efflux

## Abstract

High-density lipoproteins (HDL) maintain cholesterol homeostasis through the role they play in regulating reverse cholesterol transport (RCT), a process by which excess cholesterol is transported back to the liver for elimination. However, RCT can be altered in the presence of cardiovascular risk factors, such as aging, which contributes to the increase in the incidence of cardiovascular diseases (CVD). The present study was aimed at investigating the effect of extra virgin olive oil (EVOO) intake on the cholesterol efflux capacity (CEC) of HDL, and to elucidate on the mechanisms by which EVOO intake improves the anti-atherogenic activity of HDL. A total of 84 healthy women and men were enrolled and were distributed, according to age, into two groups: 27 young (31.81 ± 6.79 years) and 57 elderly (70.72 ± 5.6 years) subjects. The subjects in both groups were given 25 mL/d of extra virgin olive oil (EVOO) for 12 weeks. CEC was measured using J774 macrophages radiolabeled with tritiated cholesterol ((^3^H) cholesterol). HDL subclass distributions were analyzed using the Quantimetrix Lipoprint® system. The HDL from the elderly subjects exhibited a lower level of CEC, at 11.12% (*p* < 0.0001), than the HDL from the young subjects. The CEC of the elderly subjects returned to normal levels following 12 weeks of EVOO intake. An analysis of the distribution of HDL subclasses showed that HDL from the elderly subjects were composed of lower levels of large HDL (L-HDL) (*p* < 0.03) and higher levels of small HDL (S-HDL) (*p* < 0.002) compared to HDL from the young subjects. A multiple linear regression analysis revealed a positive correlation between CEC and L-HDL levels (*r* = 0.35 and *p* < 0.001) as well as an inverse correlation between CEC and S-HDL levels (*r* = −0.27 and *p* < 0.01). This correlation remained significant even when several variables, including age, sex, and BMI as well as low-density lipoprotein cholesterol (LDL-C), high-density lipoprotein cholesterol (HDL-C), and glucose levels (β = 0.28, *p* < 0.002, and β = 0.24, *p* = 0.01) were accounted for. Consuming EVOO for 12 weeks modulated the age-related difference in the distribution of HDL subclasses by reducing the level of S-HDL and increasing the level of intermediate-HDL/large-HDL (I-HDL/L-HDL) in the elderly subjects. The age-related alteration of the CEC of HDL was due, in part, to an alteration in the distribution of HDL subclasses. A diet enriched in EVOO improved the functionality of HDL through an increase in I-HDL/L-HDL and a decrease in S-HDL.

## 1. Introduction

Cardiovascular disease (CVD) is one of the major causes of mortality and morbidity in developed, middle, and low income countries [1]. The incidence of atherosclerotic cardiovascular disease (ASCVD) increases dramatically with advancing age and constitutes the main cause of mortality and morbidity in the elderly [2]. The introduction of statins some 40 years ago significantly reduced the incidence of ASCVD. However, statins have some limitations related to the side effects associated with their use and the extent to which low-density lipoproteins (LDL) levels can be safely lowered [3,4]. Moreover, despite the marked reduction in LDL cholesterol induced by statins, a significant residual cardiovascular risk was reported in several major statin therapy trials [5].

An increase in high-density lipoproteins (HDL) concentrations was proposed as an additional strategy to reduce the incidence of ASCVD. Epidemiological studies showed that there is an inverse correlation between plasma HDL levels and ASCVD risk [6]. However, pharmacological interventions aimed at increasing HDL levels were disappointing in terms of cardiovascular protection and have raised doubts about the relevance of HDL as an ideal target for atheroprotective therapies [7]. Nevertheless, this approach led to the emergence of the concept that the functionality or quality of HDL is much more important for some patients than HDL levels with respect to cardiovascular protection [8].

HDL are macromolecular complexes composed of different subclasses. Several techniques were developed to distinguish or separate the different HDL subclasses according to electrophoretic mobility, size, apoprotein content, or density. The separation of HDL by density gradient ultracentrifugation makes it possible to discriminate two main subclasses: (1) small, dense, and relatively cholesterol-poor particles, classified as HDL_3_ (7.8–8.8 nm), and (2) large, light, and relatively cholesterol-rich particles, classified as HDL_2_ (8.8–12 nm) [9]. However, there does not appear to be any significant differences between these two HDL subclasses in terms of their cardioprotective effect [10]. Nuclear magnetic resonance spectroscopy makes it possible to distinguish three HDL subclasses: small (7.3–8.2 nm), intermediate (8.3–9.3 nm), and large (9.4–14.0 nm) HDL particles. The subclasses of large HDL (L-HDL) have an inverse relationship with cardiovascular risk. Conversely, a high level of small HDL (S-HDL) particles is associated with an increased CVD risk [11,12,13]. However, the mechanisms regulating this difference between HDL subclasses remain to be elucidated.

Several cardioprotective properties are ascribed to HDL, including cellular cholesterol homeostasis and anti-inflammatory and antioxidant activities. The former is attributed to reverse cholesterol transport (RCT), a pivotal HDL pathway whereby excess cholesterol from peripheral cells, such as arterial wall cells, is transported back to the liver to be excreted in the bile or metabolized into bile salts before excretion [14]. RCT consists of three steps: (1) cholesterol efflux, where excess cholesterol is removed from cells, (2) lipoprotein remodeling, which is accompanied by structural modifications of HDL that may impact their functionality, and (3) hepatic cholesterol uptake and final excretion in bile and feces. HDL cholesterol efflux constitutes the first and most likely rate-limiting step of the RCT process. Cholesterol efflux capacity (CEC) is considered to be a metric of HDL functionality and a biomarker for the regulation of RCT [15].

Several studies have shown that there is an inverse correlation between the CEC of HDL and early risk markers for subclinical atherosclerosis and the incidence of cardiovascular events [15,16,17]. This anti-atherogenic activity of HDL is reduced in the elderly, even in the absence of the usual risk factors, which may explain, at least in part, the high incidence of CVD in the elderly. At the same time, intervention studies showed that adherence to a Mediterranean-type diet provided significant benefits in terms of cardiovascular protection, whether for primary or secondary prevention. However, the effect of this type of diet on HDL functionality and, more particularly, in the elderly remains to be elucidated.

Extra virgin olive oil (EVOO), one of the major components of Mediterranean diet, is associated with protection against cardiovascular risk factors [18] and this beneficial effect of EVOO is still significant even among persons at high cardiovascular risk [19]. The atheroprotective effect of EVOO is generally attributed to the antioxidant potential of its polyphenolic compounds, particularly hydroxytyrosol, tyrosol, and oleuropein [20]. However, few studies have investigated the effect of EVOO intake on HDL metabolism and on the parameters that may regulate the capacity of HDL in maintaining cellular cholesterol homeostasis. Our study, which is quasi-experimental one (before and after design), was aimed at investigating the effect of EVOO intake on the CEC of HDL and to elucidate the mechanisms by which EVOO intake improves the anti-atherogenic activity of HDL.

## 2. Materials and Methods

### 2.1. Chemicals

Sodium dodecyl sulfate (SDS), ethylenediaminetetraacetic acid (EDTA), and polyethylene glycol (PEG) were purchased from Sigma-Aldrich (Oakville, ON, Canada). Bovine serum albumin (BSA), fetal bovine serum (FBS), Dulbecco’s modified eagle medium (DMEM), Roswell Park Memorial Institute (RPMI) 1640 medium, and penicillin/streptomycin were purchased from Wisent (St-Bruno, QC, Canada). J774 macrophage-like cells were obtained from the American Type Culture Collection (ATCC, Manassas, VA, USA). Bio-Rad Protein Assay kits were obtained from Bio-Rad Laboratories (Mississauga, ON, Canada). Lipoprint^®^ HDL kits were obtained from Quantimetrix (Redondo Beach, CA, USA). Extra virgin olive oil (EVOO) was obtained from Atlas Olive Oils sarl (Casablanca, Morocco).

### 2.2. Extra Virgin Olive Oil (EVOO) Intake and Study Procedure

Eighty-four healthy men and women, aged between 23 and 85 years, were enrolled and distributed into two groups: 28 young (23–45 years) and 56 elderly (65–85 years) healthy subjects per group. All participants had a normal blood pressure and serum lipid profile. They were all non-smokers and not taking medication, including lipid-lowering drugs or antioxidant supplements. Pre- and postmenopausal women were included and none of the women in the elderly group were taking estrogen replacement therapy for menopause. 

The subjects were invited to consume 25 mL/d of raw EVOO for 12 weeks. Blood tests were completed at enrollment (T0) and after 12 weeks of EVOO intake (T12). The chemical composition of EVOO used in the present study is described in Supplementary Table S1 (Supplementary data) [21]. The participants were asked to maintain the same eating habits and the same level of physical activity throughout the study.

The present study was conducted according to the guidelines set out in the Declaration of Helsinki. The protocol was approved by the Ethics Committee of the Sherbrooke University Institute of Geriatrics (# 2009/19). Written informed consent was obtained from all subjects.

### 2.3. Blood Collection and Lipid Profile Determination

Fasting blood samples from the subjects were collected in EDTA and heparin blood collection tubes to determine low-density lipoprotein cholesterol (LDL-C), high-density lipoprotein cholesterol (HDL-C), total cholesterol (Total-C), triglyceride (TG), and glucose levels at T0 and T12. The blood was centrifuged to obtain plasma, with one part of plasma used to isolate HDL for CEC measurement, while the other part was stored at −80 °C until used to analyze the distribution of HDL subclasses.

### 2.4. Isolation of HDL (apoB Depleted Serum)

Plasma samples containing EDTA from the elderly subjects were immediately used to isolate HDL (at T0 and at T12) using the polyethylene glycol (PEG) precipitation method. The plasma and PEG solution (20% v/v, MW 5000–7000) were incubated for 20 min at room temperature. The mixture was centrifuged at 2000× *g* for 20 min at 4 °C and the supernatant (HDL) was then collected. Total protein concentration of HDL was determined using the Bio-Rad protein assay kit with spectroscopy (595 nm) measurement. HDL concentrations were expressed in total protein concentration.

### 2.5. Quantification of the Distribution of HDL Subclasses

HDL subfractions were measured using a Quantimetrix Lipoprint® system HDL subfractions kit according to the manufacturer’s instructions. Briefly, 25 µL of plasma sample from each subject were placed on the upper part of individual high-resolution 3% polyacrylamide Lipoprint® HDL gel tubes. The gels were photopolymerized at room temperature for 30 min. The electrophoresis was performed using a 3 mA current per gel tube at 500 V for 55 min, after which the tubes were left to rest for 30 min. The tubes were then scanned, and the digitized images were analyzed with Lipoware software. For the determination of the amount of cholesterol (mg/dL) in each band, the HDL-C concentration of the sample was multiplied by the relative area of each HDL subfraction. This method allowed us to identify 10 HDL subfractions, which could be regrouped into three subclasses: large HDL (L-HDL), medium HDL (I-HDL), and small HDL (S-HDL) particles corresponding, respectively, to subfractions 1 to 3, subfractions 4 to 7, and subfractions 8 to 10. L-HDL and I-HDL/S-HDL were also defined as fractions with densities comprised between 1.063–1.125 g/mL and 1.125–1.21 g/mL, respectively [22,23].

### 2.6. Measurement of Cholesterol Efflux

The cholesterol efflux measurements were based on a previously described method [24]. Briefly, J774 macrophages were incubated in fresh growth medium (1% FBS) and 1 μCi/mL of (^3^H) cholesterol for 24 h. The labelled cells were washed and equilibrated in a serum-free medium (1% BSA) for 12 h. They were then washed and incubated for 24 h with or without HDL (50 µg/mL) from individual subjects. At the end of the incubation period, the growth medium was recovered and centrifuged to remove cell debris. The cells were washed and thereafter lysed in PBS containing 1 M NaOH. The (^3^H) cholesterol levels were measured in the medium and the cellular lysates using a liquid scintillation counter (LS6500 multipurpose scintillation counter, Beckman, FL USA). The level of cholesterol efflux (expressed in percentage) was calculated using the following formula:

(cpm in medium/(cpm in medium + cpm in cellular lysates)) × 100

where the amount of radioactivity was expressed as counts per minute (cpm).

### 2.7. Statistical Analysis

Data are expressed as mean ± standard deviations (SD). A Student’s t-test and nonparametric tests were used when appropriate to determine statistically significant differences between the young and elderly group results. An ANOVA (Analysis of variance), followed by a Tukey multiple comparisons test, was used to compare more than two groups. Pearson’s correlation coefficient was used to assess the correlation between CEC, age, S-HDL, and L-HDL after adjusting for possible confounding variables (e.g., age, sex, body mass index (BMI), and blood pressure, etc.). The statistical analyses were performed using GraphPad Prism v9.0 (GraphPad Software, Inc. San Diego, CA, USA). For all analyses, *p* < 0.05 was considered statistically significant.

## 3. Results

### 3.1. Characteristics of the Subjects at Recruitment and after 12 Weeks of EVOO Intake

A total of 84 healthy individuals were recruited and asked to consume EVOO (25 mL/day) for 12 weeks. The main biochemical and anthropometric data of all the subjects are presented in Table 1 and Supplementary Table S2 (Supplementary data). The subjects were separated into two groups according to age: young (mean age: 31.81 ± 6.79 years) and elderly (mean age: 70.72 ± 5.6 years) (*p* < 0.001). The elderly subjects presented slightly, but significantly higher, BMIs (*p* < 0.03); higher total cholesterol (*p* < 0.001), LDL-C (*p* < 0.001), and triglyceride (*p* < 0.03) levels; higher glycemic index; and higher systolic and diastolic blood pressures (*p* < 0.03) at baseline compared to the young subjects. These values were, however, within physiological ranges. EVOO consumption for 12 weeks resulted in a trend toward a lower mean for several of the parameters, including total cholesterol, LDL-C, and triglycerides as well as systolic and diastolic pressures. However, these decreases were not statistically significant (Table 1).

### 3.2. Effect of 12 Weeks of EVOO Intake on HDL Cholesterol Efflux Capacity (CEC)

**Figure 1:**
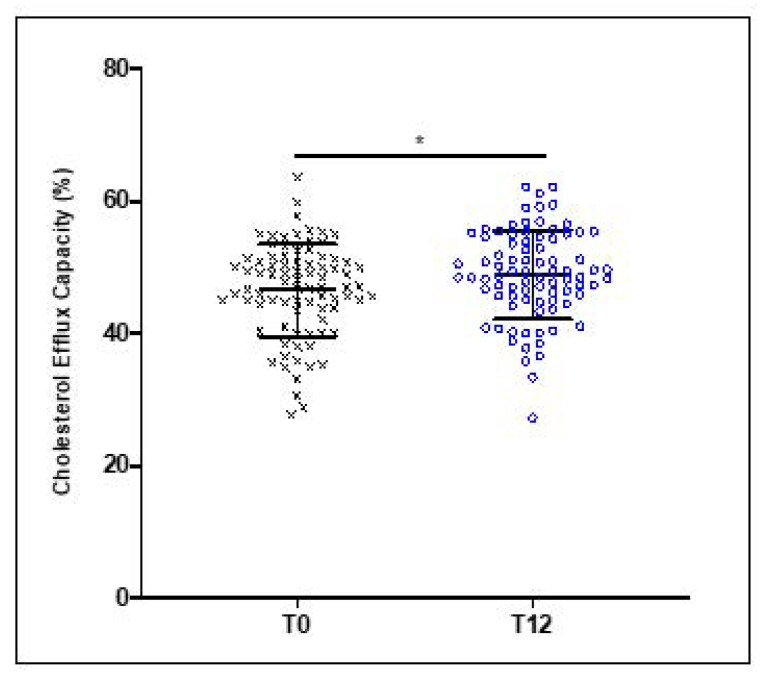
Age-related decrease in the cholesterol efflux capacity of HDL. HDL were isolated from healthy young and elderly subjects and were incubated with (^3^H)-cholesterol-loaded J774 macrophages for 24 h. The CEC of HDL was determined by measuring the percentage of radiolabeled cholesterol transferred to HDL and calculated as (cpm in medium/(cpm in medium + cpm in cellular lysates)) × 100. Results are expressed as mean ± SD (*n* = 27 young and *n* = 57 elderly healthy subjects). * *p* < 0.029.

Although no significant changes were observed for HDL levels between baseline and after 12 weeks of EVOO consumption for all 84 subjects, the CEC of HDL was significantly improved by 7.12% (*p* < 0.03) compared to the baseline values (Figure 1).

**Figure 2:**
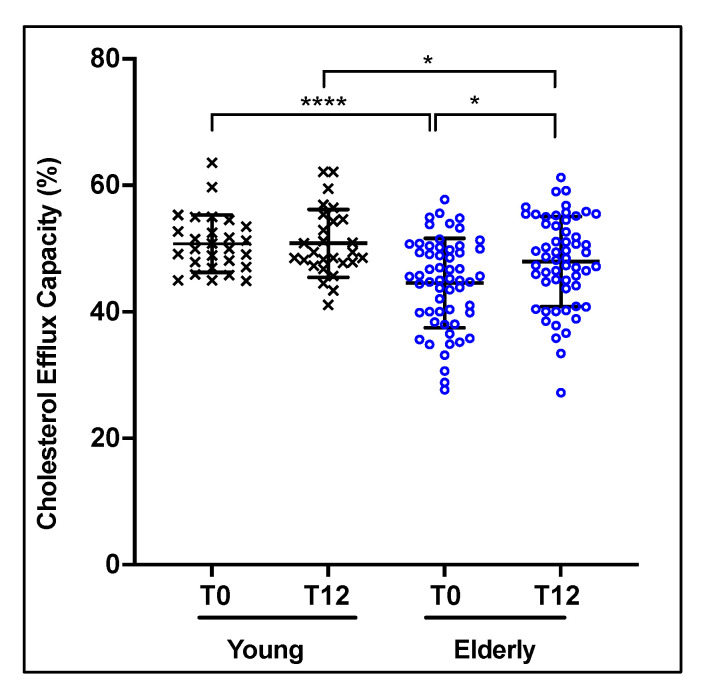
EVOO intake improves the CEC of HDL from healthy elderly subjects. Healthy young and elderly subjects consumed EVOO for 12 weeks. The CEC of HDL from each age group (young and elderly) were evaluated. HDL were incubated for 24 h with (^3^H)-cholesterol-loaded J774 macrophages. The CEC of HDL was determined by measuring the percentage of radiolabeled cholesterol transferred to HDL and calculated as (cpm in medium/(cpm in medium + cpm in cellular lysates)) × 100. Results are expressed as mean ± SD (*n* = 27 young and *n* = 57 elderly healthy subjects). * *p* < 0.03 and **** *p* < 0.0001.

Given the well-known cardioprotective effect of HDL, and the results of previous studies which suggested that the anti-atherosclerotic activities of HDL, in particular the capacity to mediate cholesterol efflux, were altered during aging, we compared the activity of HDL at baseline (T0) and after 12 weeks (T12) of EVOO consumption for the young and elderly subjects. At baseline, HDL from the older subjects exhibited a lower capacity to mediate cholesterol efflux (11.12% lower, *p* < 0.0001) compared to HDL from the young subjects (Figure 2). Interestingly, this difference in the CEC of HDL from the young and elderly subjects was reduced after 12 weeks of EVOO consumption (Figure 2). The CEC of HDL from the elderly subjects increased by approximately 8% (*p* < 0.02) after 12 weeks of EVOO consumption, whereas no significant change in the CEC of HDL from the young subjects was observed between baseline and after 12 weeks of EVOO intake (Figure 2). However, it should be noted that although the CEC of HDL from the elderly was increased following the EVOO intake, it remained significantly lower (6.91%, *p* < 0.03) when compared to that of the HDL from young subjects subjected to the same diet (Figure 2).

**Figure 3:**
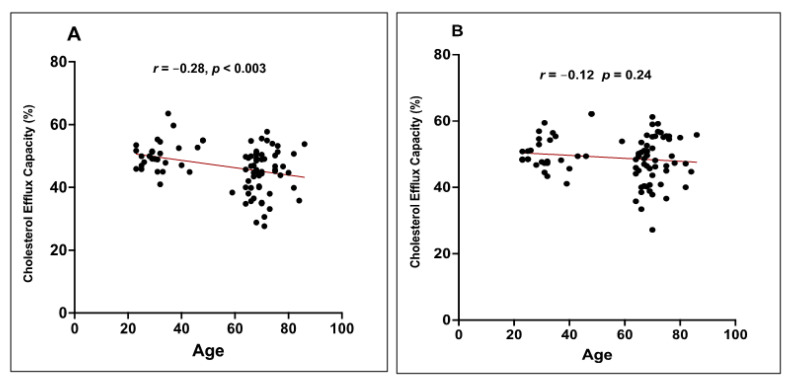
Effect of EVOO consumption on the correlation between age and the CEC of HDL. Healthy young and elderly subjects consumed EVOO for 12 weeks. The CEC of HDL from each age group (young and elderly) were evaluated for their CEC. The CEC of HDL was measured at T0 (**A**) and T12 (**B**). A Pearson’s correlation analysis was performed to determine the correlation between age and the CEC of HDL before and after EVOO consumption.

A Pearson’s correlation analysis showed that the CEC of HDL at baseline was significantly and negatively associated with the age of subjects (*r* = −0.28 and *p* < 0.003) (Figure 3A). However, this correlation disappeared after 12 weeks of EVOO consumption (*r* = −0.012 and *p* < 0.24) (Figure 3B).

### 3.3. Effect of 12 Weeks of EVOO Intake on the Distribution of HDL Subclasses

**Figure 4:**
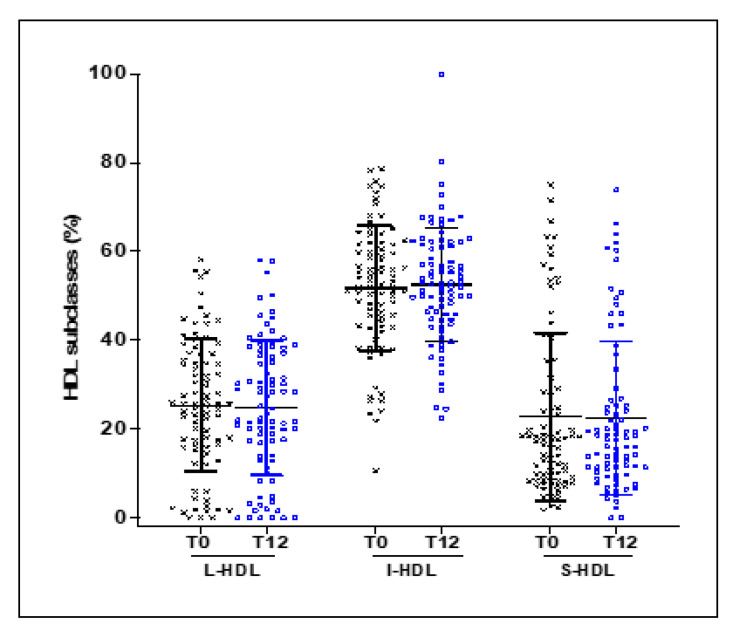
Effect of EVOO intake on the distribution of HDL subclasses. Plasma from 84 healthy young and elderly subjects was subjected to electrophoresis in 3% polyacrylamide Lipoprint® HDL gel tubes. The gel tubes were analyzed using Lipoware software. HDL was divided into 10 subfractions, which are presented here as L-HDL particles (subfractions 1 to 3), I-HDL particles (subfractions 4 to 7), and S-HDL particles (subfractions 8 to 10).

To elucidate the mechanisms that may explain the improvement in the CEC of HDL in response to EVOO intake, despite the absence of any change in plasma HDL concentrations, we investigated the effect of EVOO intake on the distribution of HDL subclasses. Plasma from each subject was subjected to electrophoresis in non-denaturing polyacrylamide gradient gels using the Lipoprint system. The distribution of the HDL subclasses showed that L-HDL, I-HDL, and S-HDL made up 23.9% ± 14.95%, 52.52% ± 13.10%, and 22.7% ± 18.99%, respectively, of the total HDL (Figure 4). No significant changes were observed between baseline and after 12 weeks of EVOO intake (Figure 4).

**Figure 5:**
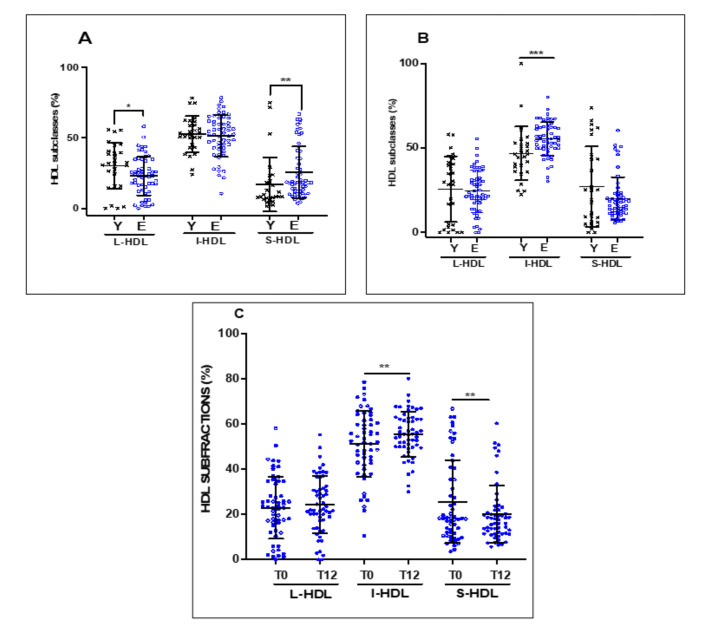
Effect of age and EVOO intake on the distribution of HDL subclasses. Plasma from healthy young subjects was obtained at recruitment and after EVOO intake. The plasma was subjected to electrophoresis in 3% polyacrylamide Lipoprint® HDL gel tubes. The gel tubes were analyzed using Lipoware software. Distribution profiles of HDL subclasses (**A**) at baseline, (**B**) after 12 weeks of EVOO intake for both young and elderly, and (**C**) for elderly only. Results are expressed as mean ± SD (*n* = 27 healthy young and *n* = 57 healthy elderly subjects). * *p* < 0.03, ** *p* < 0.002, and *** *p* < 0.0004.

We then investigated the effect of EVOO intake as a function of the age of the participants (young vs. elderly) on the distribution of the HDL subclasses. Figure 5 shows the distribution of HDL subclasses of the young subjects compared to the elderly subjects at baseline (Figure 5A) and after 12 weeks of EVOO intake (Figure 5B). There was a significant difference in the distribution of HDL subclasses at baseline between the young and elderly subjects, with the elderly subjects exhibiting higher levels of S-HDL (*p* < 0.002) and lower levels of L-HDL (*p* < 0.03) compared to the young subjects (Figure 5A). No difference was observed between the young and elderly subjects in terms of I-HDL. Interestingly, EVOO intake for 12 weeks reduced the difference between the young and elderly subjects with respect to the distribution of the S-HDL and L-HDL subclasses (Figure 5B). EVOO intake by the elderly subjects decreased their levels of S-HDL and increased their levels of L-HDL to levels equivalent to those of the young subjects. The levels of I-HDL were also significantly increased after 12 weeks of EVOO intake (Figure 5C). However, this effect was limited to the elderly subjects, and no significant changes were noted for the distribution of the HDL subclasses of the young subjects. Interestingly, the percentage of I-HDL in the elderly subjects following 12 weeks of EVOO intake increased significantly compared to the percentage of I-HDL in the young subjects (*p* < 0.0004).

### 3.4. Correlation between the Distribution of HDL Subclasses and the CEC of HDL

**Figure 6:**
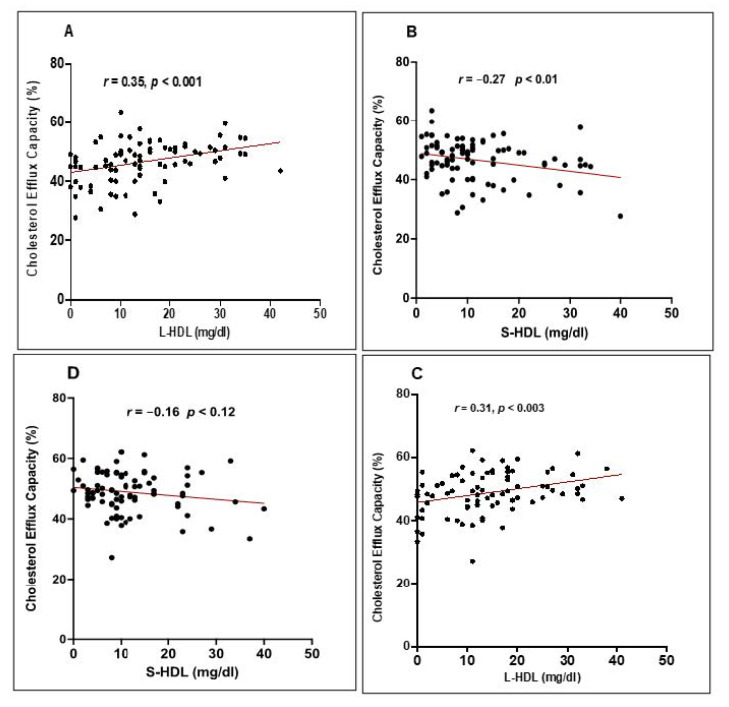
Correlation between HDL subclasses and CEC. Correlation between (**A**) L-HDL and (**B**) S-HDL and the CEC of HDL at baseline, and between (**C**) L-HDL and (**D**) S-HDL and the CEC of HDL after EVOO intake. The HDL subclass distribution was determined using Quantimetrix Lipoprint® HDL test kits. The CEC of HDL was expressed as the percentage of radiolabeled cholesterol transferred from (^3^H) cholesterol-loaded J774 macrophages to HDL calculated as (cpm in medium/(cpm in medium + cpm in cellular lysates)) × 100.

To determine whether the improvement in the CEC of HDL following 12 weeks of EVOO intake may be due to a change in HDL levels or in the distribution of HDL subclasses, we investigated the correlation between the CEC of HDL and HDL levels as well as the distribution of each HDL subclass. The correlational analysis did not reveal any relationship between HDL levels and CEC for either the young or the elderly subjects or for the entire 84 subject cohort either at baseline or after 12 months of EVOO intake (result not shown). However, the CEC of HDL was positively and significantly correlated with L-HDL levels (Figure 6A) (*r* = 0.35 and *p* < 0.001) and negatively and significantly correlated with S-HDL levels (*r* = −0.27 and *p* = 0.01) (Figure 6B). The correlation between the CEC of HDL and L-HDL levels remained significant after 12 weeks of EVOO intake (*r* = 0.31 and *p* < 0.001), but was no longer significant for S-HDL levels (Figure 6C,D). No relationship was observed between I-HDL levels and the CEC of HDL either at baseline or after 12 weeks of EVOO intake.

A multiple linear regression was performed to determine the influence of age, sex, BMI, systolic BP, diastolic BP, TG levels, LDL-C, HDL-C, and blood glucose on the correlation between the CEC of HDL and the distribution of HDL subclasses. Our results showed that the positive correlation between CEC and L-HDL, on the one hand, and the negative correlation between CEC and S-HDL, on the other, remained significant, even after adjusting the results for the different variables (β = 0.28, *p* < 0.002 and β = −0.24, *p* = 0.01, respectively) (Table 2).

## 4. Discussion

The development of atherosclerosis and the incidence of its risk factors and clinical manifestations increase dramatically with age and are responsible for most cardiovascular morbidity and mortality in the elderly. According to the Framingham risk score, the risk of developing CVD within ten years increases by one point every five years [25]. This marked impact of age on cardiovascular health is due to the multiplication and severity of the various risk factors that occur with age, particularly obesity, arterial hypertension, glucose intolerance, and dyslipidemia. However, in the absence of risk factors, other age-related biochemical and metabolic changes may also contribute to the acceleration of the atherosclerotic process in the elderly. Of these, impaired cholesterol metabolism, particularly that of HDL-C, and functionality appeared to be linked to an increase in cardiovascular risks in the elderly [26]. The present study was aimed at investigating the effect of EVOO intake on the CEC of HDL during aging and to elucidate the mechanisms by which EVOO intake improved this anti-atherogenic activity of HDL.

We evaluated the functionality of HDL by measuring the capacity of HDL to mediate cholesterol efflux, which is a metric of HDL functionality and a rate-limiting step of the RCT process. Previous studies showed that CVD is associated with an alteration in the CEC of HDL [15] and a change in the distribution of HDL subclasses, characterized by a decrease in HDL_2_ and an increase in HDL_3_ [27]. Our results showed that the HDL cholesterol levels of both the young and elderly subjects were within normal physiological values, and that HDL from the elderly subjects exhibited an impairment of their capacity to mediate cholesterol efflux from macrophages. Previous studies showed that the CEC of HDL decreased in the elderly, and have attributed this decrease to age-related oxidative stress conditions that, on one hand, induced oxidative damage to apoA-1 and, on the other hand, affected the physicochemical properties of HDL (alteration of HDL phospholipid monolayer fluidity) [28,29]. In the present study, we did not measure the oxidative status of HDL, but instead focused on the distribution of HDL subclasses.

Our results showed that HDL from the healthy elderly subjects in our cohort had higher S-HDL and lower L-HDL levels than the young subjects. This change in the distribution of HDL subclasses could be explained by the biochemical changes that occur during aging. Numerous studies showed that HDL from elderly people were characterized by significant changes in their protein and lipid composition compared to HDL from younger people [30]. Indeed, HDL from elderly people have a lower content of free and esterified cholesterol and a high content of sphingomyelin [31], and display a stiffening of their lipid layer [29]. HDL from elderly people also undergo changes in protein content, with some of the proteins being replaced by inflammatory proteins in the acute phase [32]. These changes in the composition and structure of HDL from elderly people raise the possibility that HDL particles become smaller or dysfunctional and have reduced anti-atherogenic functions [32]. This is consistent with our results showing that an increase in the level of S-HDL was associated with a reduction in the CEC of HDL.

Comparative studies of the anti-atherogenic effect of S-HDL and L-HDL have given conflicting results. An analysis of data from the Framingham study showed that plasma HDL_3_ (S-HDL) levels were associated with decreased cardiovascular risk, indicating that HDL_3_ is cardioprotective [33]. Conversely, other studies indicated that the role of HDL_3_ remained equivocal [34] and may even have a pro-atherogenic effect, whereas HDL_2_ (L-HDL) subfractions were associated with increased protection against atherosclerosis [35,36]. Lastly, some studies found no association between any of the HDL_2_ and HDL_3_ subclasses and the stage of arterial atherosclerosis [37]. Nevertheless, the present study is the first to compare the functionality of HDL with respect to their subclasses distribution as analyzed by the Lipoprint system. Interestingly, our results showed that there was a significant correlation between the CEC of HDL and the level of L-HDL. These data support the notion that L-HDL improved HDL functionality due to their anti-atherogenic property. Conversely, our results showed that there was a negative and significant correlation between CEC and S-HDL levels, suggesting that S-HDL may have a pro-atherogenic effect. The present study is the first to demonstrate that, in addition to age-related oxidative damage, the reduction in the CEC of HDL in the elderly may also be caused by an alteration in the distribution of HDL subclasses, with a decrease in L-HDL and an increase in S-HDL in elderly subjects as compared to young subjects.

Although S-HDL initiate the unidirectional transfer of cellular cholesterol via the apoA1-ABCA1 pathway, L-HDL interact with ATP binding cassette transporter A1, ATP binding cassette sub-family G member 1 and scavenger receptor class 1 (ABCA1, ABCG1, and SR-B1) and mediate the bidirectional exchange of cellular cholesterol. Consequently, a high level of S-HDL in the elderly may be the result of a weak interaction between S-HDL and peripheral cells (apolipoprotein A1 (apoA1)-ABCA1 pathway), which reduces the maturation of S-HDL to L-HDL in vivo. This is supported by the work of Sene et al., who showed that there was an age-related reduction in ABCA1 expression, which causes an impairment of senescent macrophage cholesterol efflux [38]. Higher levels of S-LDL may also be caused by a deficiency in the activity of the enzymes involved in the HDL maturation cycle or by excessive enzymatic remodeling of large HDL [39]. The reduction in L-HDL levels in the elderly may thus be a direct consequence of a decrease in the HDL maturation process.

Interestingly, our results showed that the CEC of HDL from the elderly subjects improved after 12 weeks of EVOO intake. Cholesterol efflux is a process involving the transfer of cellular cholesterol to HDL particles, which then transport the cholesterol to the liver for elimination. Cholesterol efflux is thus the fundamental process in the protection from the development of atherosclerotic lesions. Several studies showed that there is a clear association between lower cholesterol efflux and the risk of developing ACVD. Cholesterol efflux depends both on the ability of macrophages to liberate cholesterol and on the capacity of HDL to accept cholesterol. Although the liberation of cholesterol depends on the expression level of membrane cholesterol receptor (SR-BI) and transporters (ABCA1 and ABCG1), the CEC of HDL depends on the composition, physicochemical properties, and oxidative status of HDL [40]. The level of apoA-1, the composition of different phospholipids in HDL, and the fluidity of the phospholipid monolayer making up the HDL membrane are thus key factors in the regulation of this HDL activity [41]. A diet rich in olive oil can modulate the various parameters that are involved in the regulation of the CEC of HDL [41].

Although the present study was limited to investigating the link between the distribution of HDL subclasses and their CEC, changes in the biochemical composition and structure of HDL during the maturation process allow them to acquire other cardioprotective functions. HDL subclasses also differ in their antioxidant and anti-inflammatory activities. In vitro studies showed that L-HDL displayed significantly greater antioxidant activity than S-HDL [42,43] and that L-HDL have a powerful anti-inflammatory effect and may also exert an atheroprotective effect by interfering with the association of LDL with arterial proteoglycans [44]. These atheroprotective properties are a result of the biochemical changes that HDL undergo during maturation, in particular the enrichment in proteins and enzymes with immune, antioxidant, anti-inflammation, and coagulation-related properties [45]. The dynamic changes in HDL proteins define the heterogeneous functions of HDL subclasses. Although L-HDL appear to have a significantly greater atheroprotective effect than S-HDL, this does not diminish the crucial role that S-HDL play in the initiation of cholesterol exchange, which is an essential step in the formation of all HDL subclasses.

The positive correlation between the level of L-HDL and CEC and the negative correlation between the level of S-HDL and CEC indicates that L-HDL have an anti-atherogenic effect and that S-HDL have a pro-atherogenic effect. Associations between HDL size and CEC were studied, but in different contexts. In a cross-sectional study, Mutharasan et al. showed that S-HDL and L-HDL levels were negatively and positively correlated with CEC, respectively [46]. The same results were obtained in patients with hypercholesterolemia, with correlation levels comparable to ours (*r* = 0.244 for CEC and HDL-L, and *r* = 0.273 for CEC and S-HDL) [41]. This suggests, as indicated by other studies [47], that a high level of S-LDL may be considered a marker of a high risk of CVD occurring.

Our results showed that EVOO intake significantly improved the CEC of HDL in the elderly, bringing it to levels similar to that measured for HDL from young people. In addition, EVOO intake induced a change in the distribution of HDL, significantly reducing S-HDL and increasing I-HDL levels in the elderly. The level of L-LDL also increased after EVOO intake; however, this increase was not statistically significant. Our results are in agreement with those of Hernáez et al., who used randomized clinical trials to show that consuming 25 mL/day of EVOO improved the CEC of HDL [41,48]. However, the present study is the only one to show such an effect in an elderly population. Moreover, we focused on total HDL (pre-β HDL, HDL_3,_ and HDL_2_), unlike other studies that, due to the separation of HDL by ultracentrifugation, excluded the pre-β HDL fraction [41].

The improvement in CEC by HDL following EVOO intake could be explained by the physicochemical changes that occur within HDL particles following EVOO intake. Indeed, several studies showed that EVOO has a beneficial effect on various properties of HDL associated with functionality, including chemical composition and the activities of HDL-associated enzymes [41,48]. These effects were attributed to the various components of EVOO, mainly monounsaturated fatty acids and bioactive compounds, particularly polyphenols [49]. In fact, under stressful conditions, such as in inflammation, the lipid layer of HDL stiffens, which has a marked effect on their ability to promote cholesterol efflux [50]. Due to their antioxidant effect, the polyphenols in EVOO reduce lipid layer stiffening and thus improve the CEC of HDL [48].

EVOO intake is also associated with a more stable conformation of apoA-1, the main component of HDL particles, and may help explain the improvement in CEC [41]. We showed previously that phenolic compounds in EVOO induced an increase in the expression of the ABCA1 and ABCG1 cholesterol transporters in vivo. These transporters are responsible in large part for the regulation of cholesterol efflux [21]. As such, an increase in the expression of cholesterol transporters, a reduction in oxidative modifications of apoA-1, and an improvement in the composition of HDL could explain the improvement in their CEC function following EVOO intake.

EVOO intake improves the distribution of HDL subclasses, particularly in the elderly, by increasing the level of I-HDL and L-HDL and decreasing the level of S-HDL. The beneficial effect of EVOO on HDL maturation can be explained by different mechanisms, more particularly by its effect on the activity of enzymes associated with HDL, including lecithin-cholesterol acyltransferase (LCAT) and cholesteryl ester transfer protein (CETP). LCAT plays an important role in the maturation of HDL by promoting the esterification of free cholesterol, gradually converting nascent HDL (S-HDL) into mature HDL (L-HDL) [51]. Hernáez et al. showed that a Mediterranean-type, EVOO-enriched diet increased the plasma activity of LCAT [27]. LCAT is very sensitive to oxidation [52]. EVOO reduces oxidative damage to LCAT due to it high polyphenol content, thus preserving or stimulating the activity of LCAT.

Cholesteryl ester transfer protein (CETP) facilitates the transfer of cholesterol from HDL to VLDL/LDL [53], which promotes the formation of dysfunctional triglycerides-rich HDL [54]. The activity of this enzyme is high in patients at high risk for CVD [55]. The development of CETP inhibitors is currently one of the strategies being used to protect against CVD. We did not measure the CETP activity of our cohort. However, the study by Hernáez et al. showed that a Mediterranean-type diet significantly decreased the enzymatic activity of CETP [56], which supports our hypothesis of a link between LCAT/CETP enzyme activity and the distribution of HDL subclasses.

## 5. Conclusions

In summary, our results showed that there was a significant decrease in the CEC of the HDL of elderly healthy subjects. Given the importance of cholesterol efflux as the first step in the RCT process, a decrease in the CEC of HDL can have a significant impact on cellular cholesterol homeostasis and the development of the atherosclerotic process in the elderly. We also showed that the distribution profile of HDL subclasses in the elderly, even if healthy, was altered to a more pro-atherogenic HDL distribution profile (decrease in L-HDL and increase in S-HDL). Lastly, our results showed that a short 12-week intervention with an EVOO-rich diet stimulated the CEC of HDL to a level comparable to that of young, healthy subjects and improved the distribution of HDL subclasses.

## 6. Study Limitations

Our study presents two limitations: (1) although, the participants were invited to maintain the same lifestyle (nutritional habits and physical activity) throughout their participation to the study, it would have been very helpful to evaluate the nutritional differences between participants and, particularly, their intake of olive oil, regardless of the supplemented one, and to measure the levels of physical activity for each participant; and (2) the number of young people was very low when compared to elderly people and this may explain why some results were at the limit for significance. An increase in the number of participants in the young group would have allowed us to also make comparisons in each group according to sex.

## Figures and Tables

**Table 1:** Biochemical and anthropometric characteristics of the participants at recruitment and after 12 weeks of EVOO intake.

Biochemical/Anthropometric Characteristics	Young (*n* = 27)		Elderly (*n* = 57)		
	T0	T12	T0		T12
Age (years)	31.81 ± 6.79		70.72 ± 5.6		
Male	14			19	
Female	13			38	
BMI	24.37 ± 3.16	24.48 ± 3.31	26.51 ± 4.53 ^+^		26.29 ± 4.21
Systolic pressure	113.56 ± 8.43	114.40 ± 16.91	133.72 ± 18.42 ^++^		129.34 ± 15.52 **
Diastolic pressure	70.52 ± 7.84	69.71 ± 8.15	80.54 ± 8.60 ^++^		78.32 ± 7.23 **
CT (mmol/L)	4.63 ± 0.95	4.45 ± 0.81	5.52 ± 0.88 ^++^		5.45 ± 0.91 **
TG (mmol/L)	1.19 ± 0.99	1.00 ± 0.91	1.37 ± 0.74 ^+^		1.27 ± 0.66 **
HDL-C (mmol/L)	1.38 ± 0.33	1.30 ± 0.32	1.50 ± 0.40		1.53 ± 0.42
LDL-C (mmol/L)	2.70 ± 0.76	2.57 ± 0.66	3.40 ± 0.78 ^++^		3.35 ± 0.77 **
CT/HDL-C	3.62 ± 1.40	3.44 ± 1.23	3.92 ± 1.16		3.79 ± 1.08
Glucose (mmol/L)	4.18 ± 0.46	4.35 ± 0.46	4.73 ± 0.56 ^++^		4.70 ± 0.61 *

Results are presented as mean ± SD; *p*: comparison between young and elderly Mann-Whitney test, α = 0.05. **^+^** *p* < 0.03 and **^++^** *p* < 0.001 for elderly compared to young subjects at baseline; and * *p* < 0.005 and ** *p* < 0.001 after 12 weeks of EVOO supplementation. BMI: body mass index, CT; total cholesterol, TG: triglycerides, HDL-C: HDL cholesterol, LDL-C: LDL cholesterol.

**Table 2:** Multiple linear regression for correlations between CEC and L-HDL or S-HDL subclasses.

Correlations	*r*	β	*p*	*p* Adjusted
Correlation between CEC and L-HDL	0.372	0.286	0.001	0.002
Correlation between CEC and S-HDL	−0.216	−0.242	0.05	0.01

CEC: Cholesterol efflux capacity, L-HDL: Large HDL, S-HDL: Small HDL. *r*: Pearson correlation, α = 0.05.

## Data Availability

Not applicable.

## Supplementary Materials

The following are available online at https://www.mdpi.com/article/10.3390/nu13072235/s1, Table S1: chemical composition of extra virgin olive oil used in the study; Table S2: anthropometric and biochemical characteristics of the participants at baseline.
