# Supplementary material for: Extra Virgin Olive Oil Prevents the Age-Related Shifts of the Distribution of HDL Subclasses and Improves Their Functionality
Source: Nutrients. 2021 Jun 29;13(7):2235. doi: 10.3390/nu13072235 (PMC8308442; doi:10.3390/nu13072235)
Supplement: Supplementary file 1 [file nutrients-13-02235-s001.zip › nutrients-1268979-supplementary.pdf]

Supplementary Table S1. Chemical composition of extra virgin olive oil used in the study [1].

**Fatty Acids (%)**	**EVOO**
C16:0	10.4
C18:0	2.76
C18:1 n-9	71.0
C18:2 n-6	12.9
C18:3 n-3	1.04
Sterols (mg/100g oil)	319
Schottenol	0
Spinasterol	0
Stigmasta-8,22-dien-3β-ol	0
β-Sitostérol	156
Campesterol	12
Others	151
**Tocopherols (mg/Kg oil)**	257
α	190
δ	42
γ	26
**Phenolic Compounds (µg/Kg oil)**	792 983
Vanillic acid	359
Syringic acid	0
Ferulic acid	51
Tyrosol	19 573
**Others**	773 000

Supplementary Table S2. Anthropometric and biochemical characteristics of the participants at baseline.

	**Total (*n* = 84)**	**Young (*n* = 27)**	**Elderly (*n* = 57)**	
Age (years)	58.21 ± 19.22	31.81 ± 6.79	70.72 ± 5.6	<0.001
Male	35	15	20	
Female	49	12	37	
BMI	25.83 ± 4.24	24.37 ± 3.16	26.51 ± 4.53	0.033
Systolique pressure	127.32 ± 18.48	113.56 ± 8.43	133.72 ± 18.42	<0.001
Diastolique pressure	77.37 ± 9.55	70.52 ± 7.84	80.54 ± 8.6	<0.001
Total cholesterol (mmol/l)	5.23 ± 0.99	4.63 ± 0.95	5.52 ± 0.88	<0.001
Triglycerides (mmol/l)	1.31 ± 0.83	1.19 ± 0.99	1.37 ± 0.74	0.032
HDL-C (mmol/l)	1.46 ± 0.38	1.38 ± 0.33	1.5 ± 0.4	0.289
LDL-C (mmol/l)	3.16 ± 0.83	2.7 ± 0.76	3.4 ± 0.78	<0.001
CT/HDL-C	3.81 ± 1.25	3.62 ± 1.4	3.92 ± 1.16	0.113
Glucose (mmol/l)	4.56 ± 0.57	4.18 ± 0.46	4.73 ± 0.56	<0.001

Results are presented as MEAN SD, *p*: comparison between young and elderly Mann-Whitney test, $\alpha = 0.05$.

References

1. Helal, O.; Berrougui, H.; Loued, S.; Khalil, A. Extra-virgin olive oil consumption improves the capacity of HDL to mediate cholesterol efflux and increases ABCA1 and ABCG1 expression in human macrophages. *Br. J. Nutr*. **2013**, *109*, 1844–1855.
